# New Lyotropic Mixtures with Non-Chiral *N*-Acylamino Acid Surfactants Presenting the Biaxial Nematic Phase Investigated by Laser Conoscopy, Polarized Optical Microscopy and X-ray Diffraction

**DOI:** 10.3390/ma7064132

**Published:** 2014-05-27

**Authors:** Erol Akpinar, Dennys Reis, Muhammet Yildirim, Antônio Martins Figueiredo Neto

**Affiliations:** 1 Department of Chemistry, Arts and Sciences Faculty, Abant Izzet Baysal University, Bolu 14280, Turkey; E-Mail: muhammetyildirim@ibu.edu.tr; 2 Instituto de Física, Universidade de São Paulo, Caixa Postal 66318, São Paulo, SP 05314-970, Brazil; E-Mails: dennys.if@gmail.com (D.R.); afigueiredo@if.usp.br (A.M.F.N.)

**Keywords:** biaxial nematic, phase transitions, birefringence, laser conoscopy, structure, hydrogen bonding, X-ray diffraction

## Abstract

Amino acid-based surfactants were used as the main surfactants to prepare new lyotropic mixtures presenting three nematic phases. One of them is biaxial (*N_B_*), and the two others are uniaxial, discotic (*N_D_*) and calamitic (*N_C_*). These surfactants were the non-chiral molecules, potassium *N*-dodecanoyl-dl-alaninate (dl-KDDA), potassium *N*-dodecanoyl-dl-serinate (dl-KDDS), disodium *N*-dodecanoyl-dl-aspartate (dl-NaDDAs) and potassium *N*-dodecanoyl-glycinate (KDDGly). Measurements of the optical birefringences and X-ray diffraction analysis were used to characterize the nematic phases and phase transitions. Mixtures with dl-KDDS exhibited the largest biaxial phase domain (~9 °C) with respect to the other mixtures in this study. The results obtained with the KDDGly mixture showed that the existence of hydrogen bonding between the head groups of the surfactant molecules seems to hinder the orientation of the micelles under the action of an external magnetic field.

## 1. Introduction

One of the most interesting mesophases exhibited by liquid crystals is the biaxial nematic one. Yu and Saupe [1] reported the first experimental evidence of this phase in 1980, exploring the phase diagram of a ternary lyotropic mixture. They showed the existence of two uniaxial (*N_D_*, discotic; and *N_C_*, calamitic) phases and a biaxial (*N_B_*) nematic phase, confirming the theoretical predictions of Freiser [2] and Alben [3]. This fascinating field of research remains active, as evidenced by the interest demonstrated by experimentalists and theoreticians in the last decade [4,5,6,7,8].

The *N_B_* phase has been encountered in several lyotropic mixtures with, at least, a surfactant and a co-surfactant [9]. Mixtures with only one surfactant may show only one of the uniaxial nematic phases.

Lyotropic nematic mixtures doped with chiral molecules give rise to cholesteric lyotropic phases, also named lyocholesterics. Similarly to nematics, three types of lyocholesterics were identified [10,11,12]: Ch_D_, Ch_C_ and Ch_B_, where the subscripts indicate the former nematic phase, which originates the cholesteric ones. To the best of our knowledge, all the lyotropic mixtures presenting the Ch_B_ phase were obtained by the doping of nematic mixtures with brucine [13] or brucine sulfate heptahydrate [12], *i.e.*, non-amphiphilic chiral molecules. Transitions between these cholesteric phases were investigated theoretically and experimentally [13,14], showing fundamental differences with respect to the transitions in nematics. It would be interesting to investigate the transitions between the different cholesteric phases in mixtures where the main surfactant is chiral. A strategy to do that is to prepare a mixture with a racemate of the main amphiphilic molecule, *i.e.*, equal amounts of d and l enantiomers, which presents the three nematic phases. After that, changing the relative concentrations of the d and l enantiomers, the three cholesteric phases may be obtained.

Uniaxial nematic phases were obtained from mixtures prepared with some racemates [15,16]; however, the biaxial phase was not encountered in these lyotropic mixtures. We cannot discard the possibility that the *N_B_* phase exists in those mixtures, under proper conditions of temperature and relative concentrations of the components of the mixtures. However, it is important to stress that the choice of the chain length of the co-surfactant is an essential issue to be considered in the preparation of a lyotropic mixture that presents the biaxial nematic phase. Moreover, just the inspection of textures under polarizing microscope is (usually) not enough to fully characterize the nematic phase, since both the *N_B_* and *N_C_* phases show a planar texture in microslides, subjected to an external magnetic field [9]. This fact brings an ambiguity in the complete characterization of the phase and its identification as uniaxial or biaxial. To fully characterize the nematic phase, the tensor order parameter should be measured, as we will discuss in the following.

In this study, we present five new lyotropic mixtures based on *N*-acylamino acid surfactants, which present the *N_B_* phase. Four of them are composed of the racemates, potassium *N*-dodecanoyl-dl-alaninate (dl-KDDA), potassium *N*-dodecanoyl-dl-serinate (dl-KDDS) and disodium *N*-dodecanoyl-dl-aspartate (dl-NaDDAs), as the main amphiphile in each mixture. The fifth *N*-acylamino acid surfactant is the potassium *N*-dodecanoyl-glycinate (KDDGly), which is an achiral molecule. The main experimental techniques used to characterize the nematic phases are X-ray diffraction (XRD), polarized optical microscopy (POM) and laser conoscopy.

## 2. Results and Discussion

### 2.1. Nematic Phases of Racemic N-Acylamino Acid Surfactants

The labels and compositions of the samples are given in Table 2, in the Experimental Section.

The uniaxial to biaxial transition temperatures were determined analyzing the temperature dependence of the birefringences (or the symmetric invariants of the order parameter), measured with laser conoscopy and inspecting the characteristic textures in the POM. 

The order parameter that has the symmetry of the nematic phase is the optical dielectric tensor, $\overset{↔}{\text{ε}}$, a traceless second-rank tensor [2,17]. Its symmetric invariants (σ*_i_*, *i* = 1, 2, 3) may be written in terms of the optical birefringences. With the birefringences measured in the three nematic phases, the symmetric invariants of the tensor order parameter, σ_2_ and σ_3_ (σ_1_ = 0), can be calculated [18]:

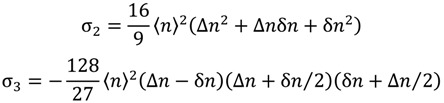

where <n> is the mean index of refraction of the mixture. In the framework of the Landau-de Gennes theory (*i.e.*, mean-field theory), these invariants show a linear dependence with the temperature in the vicinity of the second-order uniaxial to biaxial phase transition.

**Figure 1:**
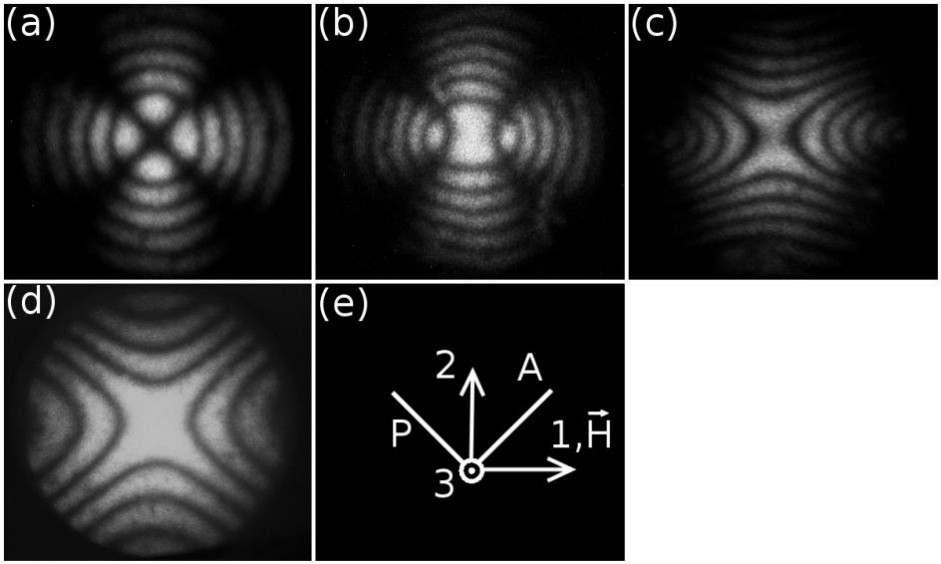
Typical conoscopic patterns of the mixture, potassium *N*-dodecanoyl-dl-serinate (dl-KDDS)/Na_2_SO_4_/1-dodecanol (DDeOH)/water. (**a**) nematic discotic phase (*N_D_*) at 45.0 °C; (**b**) nematic biaxial phase (*N_B_*) near the *N_D_* to *N_B_* transition at 33.7 °C; (**c**) *N_B_* at 31.0 °C; and (**d**) calamitic nematic phase (*N_C_*) at 23.0 °C; (**e**) geometry of the experiment: *P*, *A* and $\vec{H}$ are the directions of the polarizer, analyzer and the applied magnetic field, respectively.

In our experiments, usually, we started the measurements with the sample in the N_D_ phase, perfectly oriented with the director parallel to the laser beam. The temperature is then changed step by step to reach the other nematic phases. Figure 1 shows typical results of the conoscopic patterns obtained in our experiments.

We have checked that the phase sequence as a function of the temperature does not change (within the precision in the measurement of the temperature) for samples stored in a freezer for at least one year. The aspect of the conoscopic fringes is also the same, without noticeable modifications as a function of time. This result assures us that the patterns represent the steady state of the alignment of the sample, subjected to the magnetic field. At this point, it is interesting to discuss in more detail the features of the conoscopic patterns shown in Figure 1. The symmetry of the patterns and the contrast between the fringes and the background assures us that they were produced by well-aligned samples. Unaligned samples could not produce such types of patterns (see, e.g., [19]). Observing only the central part of the pattern (the opening of the Maltese cross), obviously, we cannot differentiate the existence of a biaxial phase from the effect of a uniaxial phase losing its alignment [20]. However, the complete conoscopic patterns of these two situations are completely different. This assures us the existence and alignment of the biaxial phase in our experiments.

**Figure 2:**
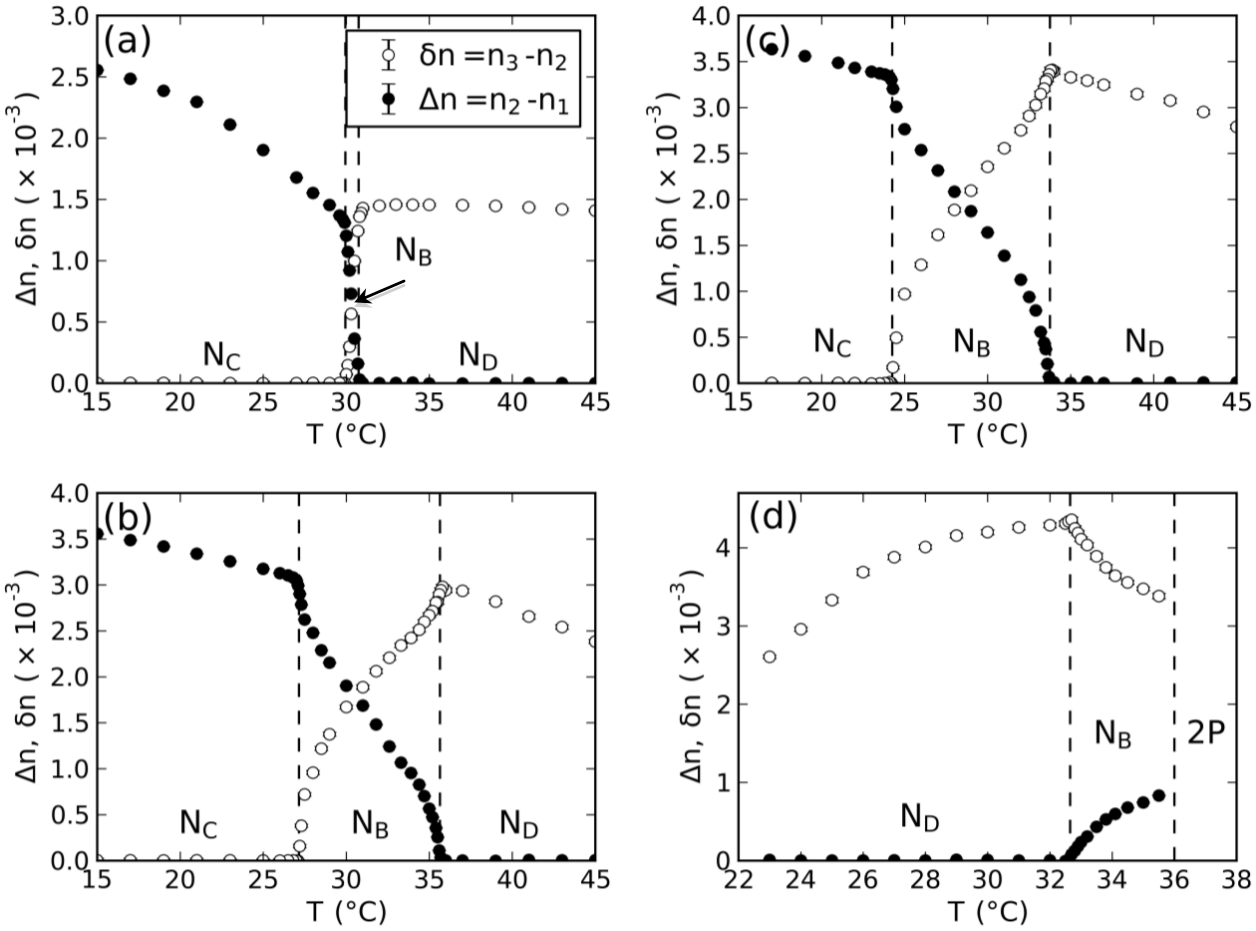
Temperature dependence of the birefringences of the mixtures: (**a**) potassium *N*-dodecanoyl-dl-alaninate (dl-KDDA)/Cs_2_SO_4_/DDeOH/water; (**b**) dl-KDDS/Na_2_SO_4_/1-undecanol (UndeOH)/water; (**c**) dl-KDDS/Na_2_SO_4_/DDeOH/water; and (**d**) disodium *N*-dodecanoyl-dl-aspartate (dl-NaDDAs)/Cs_2_SO_4_/UndeOH/water. 2P represents a two-phase coexistence region.

The temperature dependence of the birefringences of dl-KDDA, dl-KDDS and dl-NaDDAs are given in Figure 2.

Biaxial nematic domains are present in the phase sequences shown in Figure 2, in between the two uniaxial nematic phases, except in the case of the dl-NaDDAs mixture, where the *N_B_* phase domain ends in a two-phase region at higher temperatures. Interestingly, this last mixture shows the *N_D_* phase domain at lower temperatures, contrary to the other mixtures investigated, where the *N_C_* phase domain occurs at lower temperatures.

**Figure 3:**
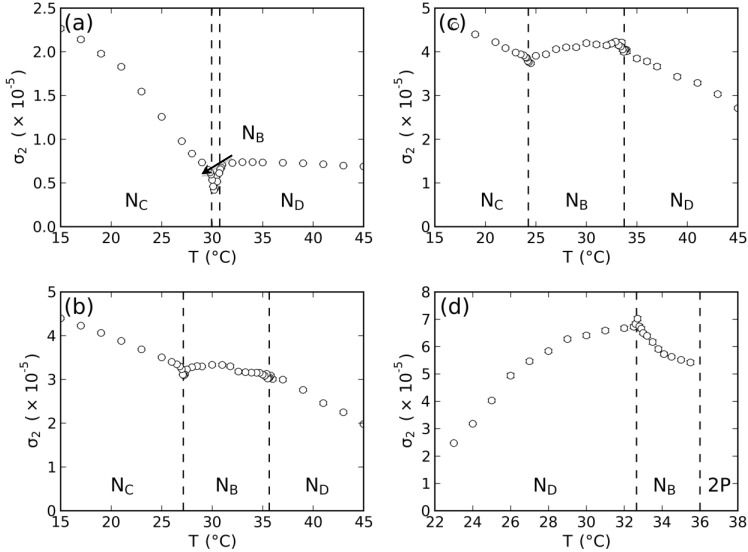
Temperature dependence of the tensor order-parameter symmetric-invariant, σ_2_, of the mixtures: (**a**) dl-KDDA/Cs_2_SO_4_/DDeOH/water; (**b**) dl-KDDS/Na_2_SO_4_/UndeOH/water; (**c**) dl-KDDS/Na_2_SO_4_/DDeOH/ water; and (**d**) dl-NaDDAs/Cs_2_SO_4_/UndeOH/water.

**Figure 4:**
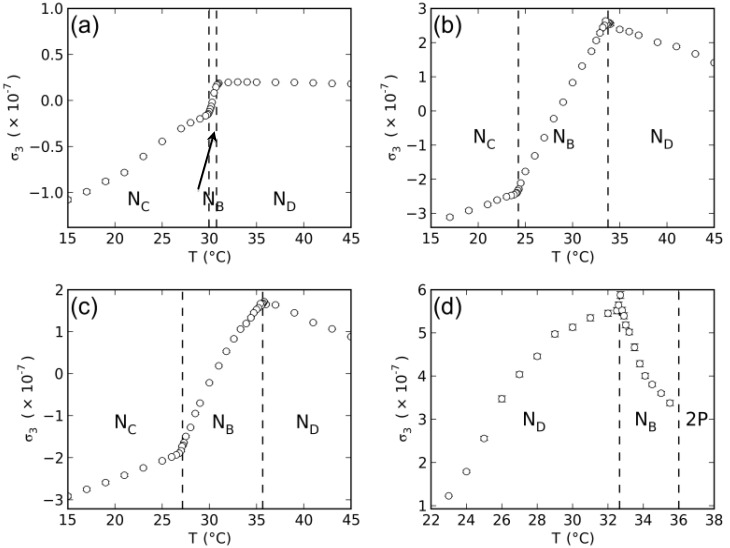
Temperature dependence of the tensor order-parameter symmetric-invariant, σ_3_, of the mixtures: (**a**) dl-KDDA/Cs_2_SO_4_/DDeOH/water; (**b**) dl-KDDS/Na_2_SO_4_/UndeOH/water; (**c**) dl-KDDS/Na_2_SO_4_/DDeOH/water; and (**d**) dl-NaDDAs/Cs_2_SO_4_/UndeOH/water.

**Figure 5:**
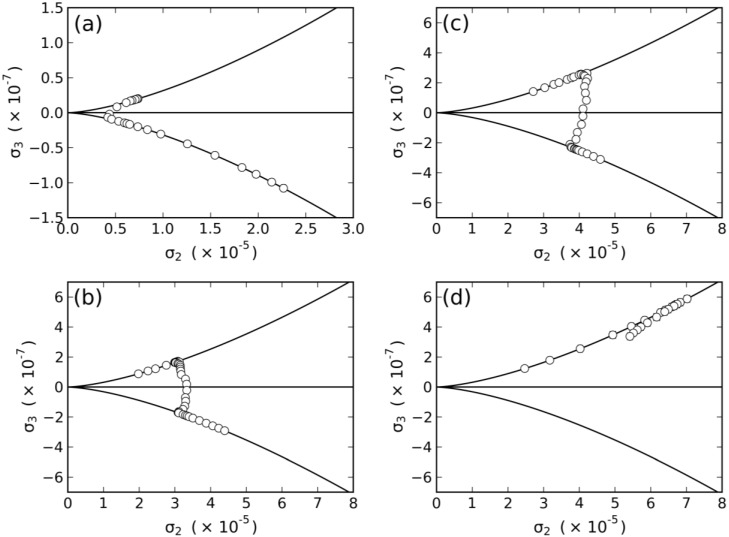
Loci of the nematic phases in the space of the invariants, σ_2_ and σ_3_, of the mixtures: (**a**) dl-KDDA/Cs_2_SO_4_/DDeOH/water; (**b**) dl-KDDS/Na_2_SO_4_/UndeOH/water; (**c**) dl-KDDS/Na_2_SO_4_/DDeOH/water; and (**d**) dl-NaDDAs/Cs_2_SO_4_/UndeOH/water.

With the measured values of the birefringences in the nematic phases, the tensor order-parameter symmetric-invariants may be calculated. Figure 3 and Figure 4 show the temperature dependence of the invariants, σ_2_ and σ_3_, respectively. The behaviors of σ_2,3_ × *T* in the vicinity of the uniaxial to biaxial nematic phase transitions are in accordance with the mean-field prediction, *i.e.*, σ_2_ and σ_3_ show a linear dependence with the temperature. Figure 5 depicts the locations of the nematic phases in the space of the invariants. Solid lines correspond to the uniaxial nematic phases: ${\text{σ}}_{3}=\pm {\text{σ}}_{2}^{3/2}$, where the + and − signs refer to the *N_D_* and *N_C_* phases, respectively.

The textures of the nematic phases of these mixtures observed in the POM are the classical ones, already reported in the literature [9]. An interesting question that may be proposed is how the d- and l-enantiomers arrange in the micelles to give rise to a non-chiral nematic phase. However, for their pure l-enantiomers, there is some experimental evidence about their organization in the chiral micelles. Du and co-workers [21] investigated the molecular arrangement of *N*-hexadecanoyl-l-alanine (*N*-HDA) by atomic force spectroscopy. This molecule belongs to the class of *N*-acylamino acids being similar to the amino acid-based surfactant molecules employed by us in this study. This molecule has three additional CH_2_ groups in its alkyl chain, with respect to our molecules, and its head group is neutral, e.g., not potassium or sodium salt. They analyzed the formation of the monolayers by chiral *N*-HDA molecules on the silicon and mica surfaces and found that the hydrocarbon chains of *N*-HDA molecules arrange as almost tilted and parallel to each other in the bilayer. They interpreted this molecular arrangement by means of the homochiral effect between chiral carbons in the head groups. A similar result was also reported for monoalkylethylenediamines [22]. It is expected that the head groups of *N*-HDA molecules, l-alanine, form hydrogen bonding among them via neighboring carboxylic acid and amide groups (–C=O···N–H) at the surface of the micelles.

**Figure 6:**
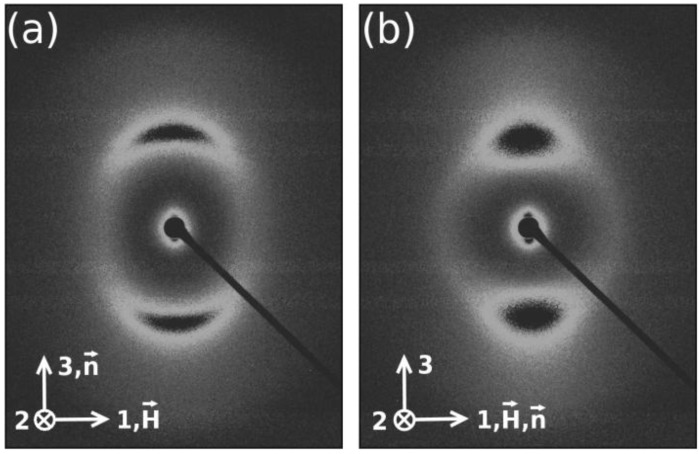
Typical X-ray diffraction patterns of the mixture dl-KDDS/Na_2_SO_4_/1-dodecanol/H_2_O: (**a**) *N_D_* phase, *T* = 36.0 °C; (**b**) *N_C_* phase, *T* = 24.0 °C.

X-ray diffraction experiments performed with these mixtures gave information about the structure and local ordering of the micelles. A difficulty we faced with mixtures where Cs ions were present was the high absorption coefficient of the mixtures for the X-ray wavelength employed in the experiments. In the particular case of Samples 1 and 4, it was not possible to have reliable diffraction patterns in our experimental setup. The data of the different samples are presented in Table 2. s*_i_*, *i* = 1,3 are the moduli of the scattering vector that correspond to the position of the diffraction bands along the horizontal (1-axis) and vertical (3-axis) of the laboratory frame axes. Thus, the repeating distances along these directions are s*_i_*^−1^. Typical X-ray patterns are presented in Figure 6. The X-ray scattering at small angles (visible near the beam stopper in Figure 6) will not be analyzed in the present work. Three bands exist in these patterns: two along the 3-axis and one along the 1-axis directions. The outer band along the 3-axis is barely visible in the patterns. 

**Figure 7:**
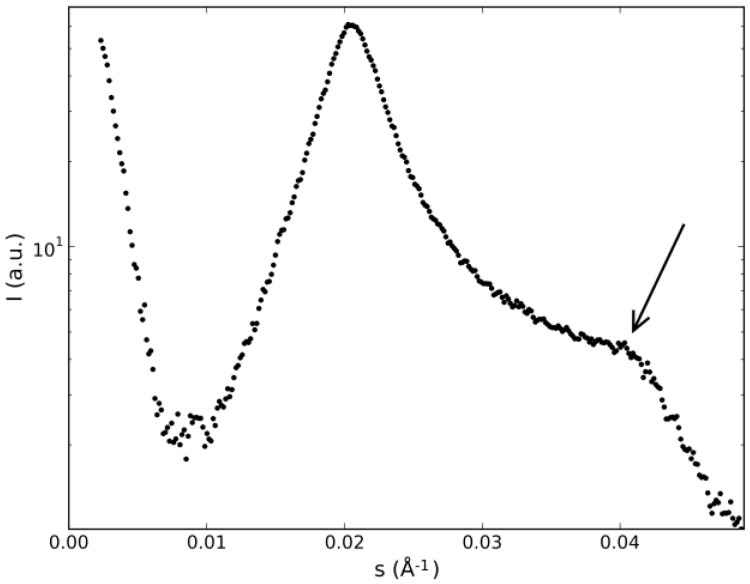
Diffracted intensity (in logarithmic scale) as a function of the modulus of the scattering vector along the 3-axis. Mixture dl-KDDS/Na_2_SO_4_/1-dodecanol/H_2_O; *N_D_* phase, *T* = 36.0 °C. The arrow indicates the location of the second-order band.

The plot of the diffracted intensity as a function of the modulus of the scattering vector along the 3-axis (Figure 7) reveals its presence. The two bands along the 3-axis are originated from the pseudo-lamellar ordering of the micelles, corresponding to the first and second-order bands, typical of lyotropic nematics with, at least, two amphiphiles [23]. To go further in the analysis of the diffraction bands, we have to assume a model for the micelles. The model that is consistent with all the results from the different experimental techniques, adequate to describe the micelles in mixtures that present the three nematic phases, is the intrinsically biaxial micelles (IBM) model (see, e.g., [9] for a comprehensive discussion about this model). This model assumes that the micelles have an orthorhombic symmetry in the three nematic phases, and orientational fluctuations of the correlation volumes originate the different symmetries of the nematic phases. The micelles may be sketched as a flattened ellipsoid, with three characteristic dimensions: the smallest one corresponds to the main amphiphilic bilayer, and the two others lie in the flat surface of the ellipsoid. Without additional information about the thickness of the main amphiphilic bilayer of the different mixtures investigated, we have to restrict our analysis to the average volume available per micelle, *i.e.*, the micelle itself and the water that surrounds it. We define a parameter that evaluates the anisotropy of the available volume per micelle as ϒ = s_1_^−1^/s_3_^−1^. It is interesting to compare the values of ϒ obtained in our present experiment with that encountered in the ternary mixture of KL/decanol/water, which is ϒ~2.2. It is expected that the bigger ϒ, the bigger the shape anisotropy of the micelle itself. Thus, the micelles present in Mixtures 2 and 3, in the nematic phases, are expected to be more symmetric (*i.e.*, less anisometric) than those on the KL mixture. This fact is consistent with the maximum values of the birefringences in these racemic mixtures, which are also smaller than those encountered in the KL mixture. Interestingly, the correlation lengths associated with the diffraction bands along Axis 3 of both racemic mixtures are smaller than those of the KL mixture (e.g., ξ_3_~30 nm); however, those along Axis 1 are bigger than that of the KL mixture (e.g., ξ_1_~6 nm). The order parameters associated with the first-order band along the 3-axis were calculated according to Deutsch calculation [24] and show values consistent with the measurements of the coherence lengths, *i.e.*, the higher ξ, the higher the order parameter.

### 2.2. Nematic Phases of KDDGly/Na_2_SO_4_/TDeOH/Water

**Figure 8:**
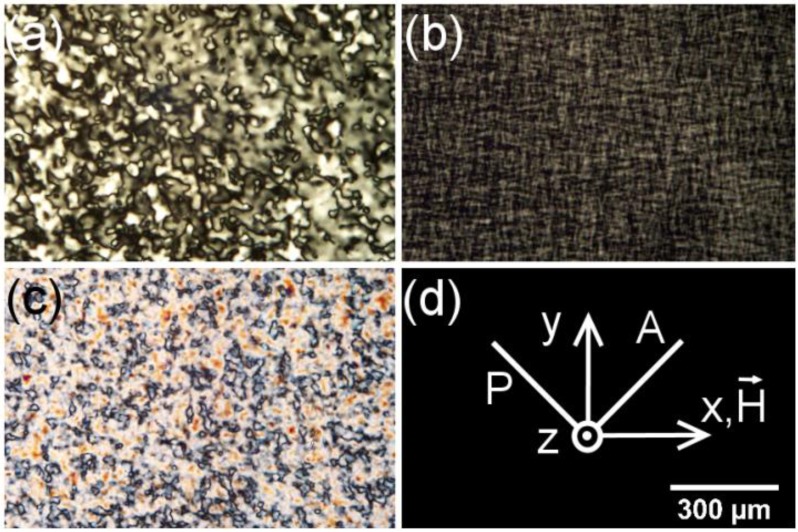
Textures of *N*-dodecanoyl-glycinate (KDDGly)/Na_2_SO_4_/1-tridecanol (TDeOH)/water mixture under a magnetic field of 0.3 kG: (**a**) *N_D_* at 40.0 °C after 1 h; (**b**) *N_D_* to *N_B_* phase transition at ~30.1 °C; and (**c**) *N_C_* at 23.00 °C; (**d**) laboratory reference frame. *P*, *A* and $\vec{H}$ are the directions of the polarizer, analyzer and magnetic field, respectively.

This new mixture presents nematic phases, but exhibited a peculiar behavior with respect to the other racemic *N*-acylamino acid surfactants mixtures in the conoscopy experiment. The *N_D_* phase aligns perfectly in the magnetic field (applying the alignment procedure previously described) only after about four hours. When the temperature is decreased, the center of the conoscopic pattern (the Maltese cross; see, e.g., Figure 1a) splits, indicating the beginning of the *N_B_* phase domain, similar to Figure 1b. Suddenly, all the fringes disappear just after the *N_D_* to *N_B_* transition, indicating a loss of the sample’s alignment. The usual orientational procedure employed to align the *N_B_* phase that was shown to be effective in the case of the other mixtures reported here was not enough to orient the biaxial nematic phase in this case. Let us describe, now, the behavior of this mixture placed in a 0.2 mm flat microslide under the POM experiment. In the *N_D_* phase, 1 h after filling the microslide and in the presence of the magnetic field, a characteristic schlieren texture was observed (Figure 8a). After about 5 h, the texture is pseudo-isotropic, with the director perpendicular to the glass surfaces of the microslide and to the magnetic field. Decreasing the temperature, the sample transits to the *N_B_* phase, and the texture reveals defects characteristic of a non-oriented sample (Figure 8b). This result explains to us the disappearance of the conoscopic fringes in the *N_D_* to *N_B_* phase transition, since the sample completely lost its alignment. Decreasing the temperature to about 23 °C, the texture observed is characteristic of the *N_C_* phase (Figure 8c).

To find the temperature of the *N_C_* to *N_B_* phase transition, we kept the sample in the microslide about 72 h at 23.0 °C, in the presence of the magnetic field. A planar texture was obtained with the director aligned parallel to the magnetic field. With the help of a Berek compensator, we measured the optical birefringence in the *N_C_* phase Δ*n* ~ 3 × 10^−3^. Slowly increasing the temperature, when the *N_C_* to *N_B_* transition is achieved (*T* = 25.8 °C), the sample lost its alignment, and the conoscopic fringes do not allow a precise measurement of the birefringence. This behavior is very similar to what we observed at the *N_D_* to *N_B_* transition. This behavior, however, is different from that observed with the racemic mixtures investigated in this work. With those racemic mixtures, when the uniaxial to biaxial phase transition takes place, the two optical axes of the *N_B_* phase lie in the plane perpendicular to the magnetic field, and those, which are not there, are easily replaced in that plane by the orientational procedure described in the Experimental Section. The KDDGly mixture, however, presents a weak coupling with the magnetic field, which avoids the quick orientation of the two optical axes of the *N_B_* phase on that plane. This weak magnetic coupling was also observed in the *N_C_* phase subjected to the magnetic field that took about three days to achieve the planar orientation of the director. Thus, when the sample transits from a uniaxial to the biaxial phase, the two optical axes, in different correlation volumes lie in different planes, giving rise to the defects observed in the textures (see, e.g., Figure 8b).

**Figure 9:**
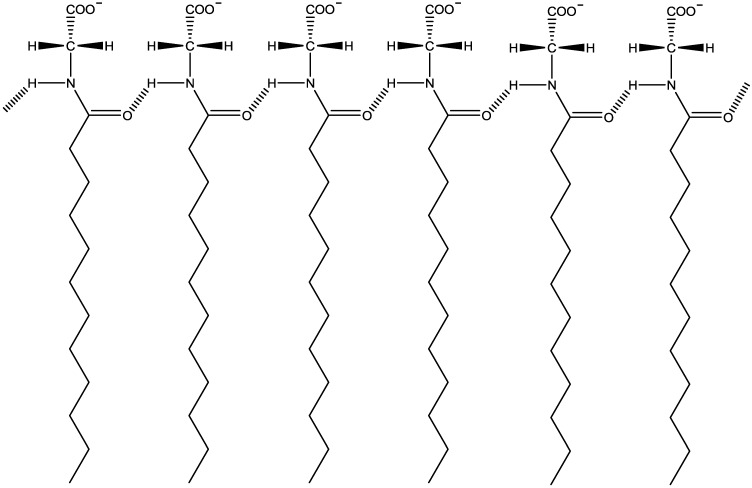
Molecular arrangement of the KDDGly molecules. The hashed bonds between –C=O and –N–H correspond to the hydrogen bonds.

Let us point out some characteristics of the KDDGly molecule that could help us understand why the nematic phases of this mixture present a weak magnetic coupling. Due to the structure of the KDDGly molecule head group, it is expected that hydrogen bonds exist between neighboring molecules in the micelles [25] (Figure 9).

Comparing the molecules KDDGly and KL, the main difference between them is that the KDDGly molecules form hydrogen bonds between them and the KL molecules do not. The different nematic phases of the largely investigated lyotropic mixture, KL/decanol/water [1,9], show an effective magnetic coupling. The orientational procedure employed by us quickly (~minutes) orients the sample. It is known that in micellar systems, there exist a characteristic time for the exchange of amphiphilic molecules between the micelles and the bulk, being of the order of s [26]. When a system composed of anisotropic micelles (as in the case of the lyotropic τ ~ 10^−5^–10^−3^s in nematic phases) is subjected to an external magnetic field, the alignment process is complex, having different contributions: (1) a sterical contribution from the micelle-micelle interaction (collective behavior); (2) the coupling of an individual micelle with the magnetic field; and (3) the coupling from each individual amphiphilic molecule (even from the bulk) with the field. The aliphatic chains of the amphiphilic molecules present the anisotropy of the diamagnetic susceptibility of the order of (−10^−4^) cgs [27]. In the presence of the magnetic field, we expect that the molecular exchange between micelles and the bulk takes the field constraint into account and that the molecules entering in the micelles, as time goes by, be placed in more energetically favored locations in terms of the magnetic coupling between molecules and the field. The existence of hydrogen bonds (typical energy of 10 kcal/mol [28]) between neighboring KDDGly molecules in the micelles could increase this typical time, τ. Coarsely, we could say that micelles with the KDDGly molecules are basic units “more rigid” with respect to those constituted by molecules that do not form hydrogen bonds. In this framework, the KDDGly system would show a lower response to the magnetic field, explaining our difficulty in orienting this sample.

The X-ray diffraction patterns of this mixture also show the pseudo-lamellar ordering, with the first and second-order bands along the 3-axis and a broad band along the 1-axis (see Table 1). The coherence lengths encountered were similar to those of the racemic mixtures. However, the order parameter calculated was smaller, which is consistent with the difficulty in obtaining a well-oriented sample under the action of a magnetic field.

**Table 1:** X-ray diffraction data. s*_i_*, *i* = 1,3 represents the moduli of the scattering vector that correspond to the position of the diffraction bands along the horizontal (1-axis) and vertical (3-axis) directions of the laboratory frame axes. OP represents the order parameter, and ξ*_i_*, *i* = 1,3 represents the correlation length corresponding to each diffraction band. ϒ = s_1_^−1^/s_3_^−1^ represents the average anisotropy of the available volume per micelle.

Sample	Phase	s_1_^−1^ (nm)	s_3_^−1^ (nm)	OP	ξ_1_ (nm)	ξ_3_(nm)	ϒ
2	*N_C_*	7.2 ± 0.3	4.9 ± 0.1	0.27	9	19	1.5
3	*N_D_*	6.5 ± 0.3	4.9 ± 0.1	0.43	12	20	1.3
5	*N_D_*	6.8 ± 0.5	5.5 ± 0.1	0.29	11	19	1.2

## 3. Experimental Section

**Figure 10:**
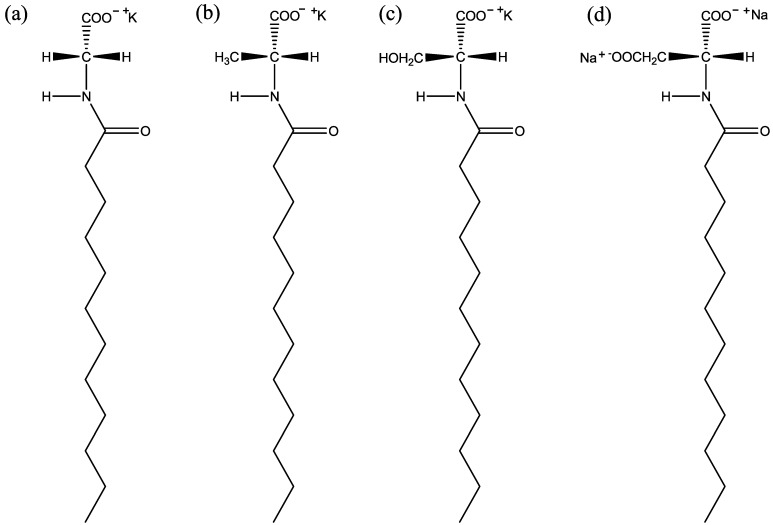
The molecular structures of *N*-dodecanoylamino acid surfactants: (**a**) KDDGly; (**b**) d-KDDA; (**c**) d-KDDS; and (**d**) d-NaDDAs. For (**b**), (**c**) and (**d**), their mirror images give corresponding l-enantiomers, *i.e*., l-KDDA, l-KDDS and l-NaDDAs, respectively. dl-racemic mixtures are composed of a 50:50 percent of d- and l-enantiomers.

Dodecanoyl chloride, dl-alanine, dl-serine, dl-aspartic acid, glycine, Na_2_SO_4_, Cs_2_SO_4_, undecanol, dodecanol and tridecanol were purchased from Merck (Darmstadt, Germany) for Merck and for Sigma), Sigma (Missouri, USA), Fluka and Alfa-Aesar with purities >99%. dl-KDDA and dl-KDDS were synthesized via Jungermann’s method [29] with a minor revision. After *N*-acylamino acids were prepared by the reaction of dodecanoyl chloride with the corresponding amino acid, they were precipitated from boiling toluene, and the products, *N*-acylamino acids, were filtered off as white solids. In this way, since dodecanoic acid, which may be produced during the amidization reaction, is highly soluble in toluene at room temperature, it can be removed from the products easily. Then, the *N*-acylamino acids were neutralized with ethanolic KOH in ethanol to obtain their potassium salts at room temperature. Since Jungermann’s method gave a very low reaction yield (<40%), we applied an alternative method to synthesis dl-NaDDAs and KDDGly given in the literature [30]. All *N*-acylamino acid surfactants were characterized by FTIR spectroscopy. The molecular structures of the surfactant molecules are given in Figure 10.

### 3.1. Sample Preparation

Lyotropic liquid crystalline mixtures were prepared by weighing each constituent in test tubes. Mixtures were subjected to successive agitation in vortex and centrifugation until complete homogenization. Heating was not necessary during the mixture preparation procedure. In the following, a water-based ferrofluid (Ferrotec) was added to the mixture (about 1 µL of ferrofluid per 1 g of the mixture). It is known that this amount of ferrofluid does not affect the phase transition temperatures and phase sequence of the mixture [9]. The mixtures concentrations are given in Table 2.

**Table 2:** Compositions of the mixtures investigated. The phase sequences correspond to the observed phases (from left to right, the temperature was increased). UndeOH, DDeOH and TDeOH are the alcohols, 1-undecanol, 1-dodecanol and 1-tridecanol, respectively. *X* is the mole percent fraction of each constituent. Δ*T*_NB_ represents the biaxial nematic phase domain.

Sample	Surfactant	*X*_surf_	*X*_Cs_2_SO_4__	*X*_Na_2_SO_4__	*X*_UndeOH_	*X*_DDeOH_	*X*_TDeOH_	*X*_H_2_O_	Phase sequence	Δ*T*_NB_ (°C)
1	dl-KDDA	3.33	0.94	–	–	1.08	–	94.64	*N_C_*, *N_B_*, *N_D_*	~1.10
2	dl-KDDS	3.56	–	1.09	0.68	–	–	94.67	*N_C_*, *N_B_*, *N_D_*	~8.60
3	dl-KDDS	3.41	–	1.15	–	0.76	–	94.67	*N_C_*, *N_B_*, *N_D_*	~9.60
4	dl-NaDDAs	3.95	3.05	–	2.05	–	–	90.95	*N_D_*, *N_B_*	~2.85
5	KDDGly	2.74	–	1.00	–	–	0.81	95.45	*N_C_*, *N_B_*, *N_D_*	~4.35

### 3.2. Laser Conoscopy

Laser conoscopy [18] is a very reliable technique to measure the two birefringences (∆*n* = *n*_2_ − *n*_1_ and δ*n* = *n*_3_ − *n*_2_) in the three nematic phases, *N_D_*, *N_B_* and *N_C_*, as a function of temperature. The subscripts, 1, 2 and 3, refer to the laboratory frame axes with respect to which of the nematic phases are aligned: the two orthogonal Axes 1 and 2 define the horizontal plane, and Axis 3 is perpendicular to this plane and parallel to the laser beam propagation direction. It is possible to precisely measure birefringences of the order of 10^−3^ with this technique, which is the case for lyotropic liquid crystals.

For birefringence measurements via laser conoscopy, lyotropic nematic samples doped with ferrofluid were transferred into a cell made of two optical circular glasses and a ring of glass 2.5 mm-thick (sample thickness set in 2.5 mm). A static magnetic field *H* = 3.05 kG (Walker Sci. electromagnet), parallel to Axis 1, helps the orientation of the samples. The experimental setup has a HeNe laser (λ = 632.8 nm), a Neocera LTC-21 temperature controller (Neocera, Beltsville, USA), with a precision of 0.001 °C, and a Julabo Refrigerated/Heating water-bath circulator (Julabo, Seelbach, Germany) with a precision of 0.01 °C.

A key point in the laser conoscopy technique is to get high-quality conoscopic patterns, which are obtained with well-aligned samples. For this purpose, samples were rotated about the angle of ±30° several times around Axis 3 in the *N_D_* and *N_B_* phases with an applied external magnetic field (*H* = 3.05 kG). Then, ongoing from *N_D_* to *N_C_*, passing through *N_B_*, the measurement of the birefringences is performed as a function of temperature. In the case of the *N_C_* phase, the sample is left at rest in the presence of $\vec{H}$ (see, e.g., [9] for details).

### 3.3. X-ray Diffraction

The microstructure and local ordering of the micelles were investigated by X-ray diffraction using a Xenocs Xeuss system, which consists of a GeniX^3D^ beam delivery system with a Cu anode X-ray tube (λ = 0.15411 nm), a collimation composed of two scatterless slits and a Pilatus 300K detector (Dectris, Baden, Switzerland). The beam has a square cross-section of 0.8 × 0.8 mm^2^. The beam center correction and the sample to detector distance of 72.6 cm were measured with a sample of silver behenate. The sample holder is a small parallelepiped made of copper with inner channels for water circulation, which enables the control of temperature with a precision of 0.1 °C. There is a centered vertical hole parallel to the long face of the parallelepiped to place glass capillary tubes of 1.5 mm in diameter. Perpendicular to this, there is a horizontal small window that allows the passage of the X-ray beam. Besides, two permanent magnets are attached to the sidewalls of the holder. The magnetic field in sample position is of about 1 kG. The samples were oriented in each nematic phase using the same procedure described above for laser conoscopy. The laboratory frame of reference was defined with Axis 3 along the vertical direction parallel to the capillary long axis, Axis 2 in the direction of the X-ray beam and Axis 1 parallel to the magnetic field direction.

Measurements in the N_C_ and N_D_ phases have been done for the different lyotropic samples. At a given temperature, the samples were irradiated in the direction perpendicular to the magnetic field direction. The exposure time for each measurement was 15 min. The data analysis was done in the framework proposed in [23], and the order parameter was calculated according the work of Deutsch [24]. The angular and radial integrations were done using the FIT2D software. Water (background) and noise subtractions were done for each integrated datum.

## 4. Conclusions

Five new lyotropic mixtures were reported presenting the biaxial nematic phase. In particular, four of them have in their composition racemates of *N*-acylamino acid surfactants as the main amphiphile. In three of these mixtures, the two uniaxial and the biaxial nematic phases were identified. The analysis of the optical birefringences and the invariants of the order parameter as a function of the temperature indicate that the uniaxial to biaxial phase transitions are of second order. The mixtures with dl-KDDS were those that presented the largest biaxial phase domain in terms of temperature. Moreover, the bigger the number of carbon atoms on the aliphatic chain of the co-surfactant, the larger the biaxial phase domain. In the case of the mixture with KDDGly, the three nematic phases were also identified. However, the orienting process that combines the presence of a magnetic field and rotations of the sample, extremely efficient in all the other mixtures investigated here, was shown to be not efficient. In the uniaxial phases, the orientation was achieved after a long time, and in the biaxial phase, it was not even completely achieved. Hydrogen bonds between neighboring head group molecules of KDDGly are suggested as being responsible for this weak response of the system to the magnetic field.

## References

[1] Yu L.J., Saupe A. (1980). Observation of a Biaxial Nematic Phase in Potassium Laurate-l-Decanol-Water Mixtures. Phys. Rev. Lett..

[2] Freiser M.J. (1970). Ordered States of a Nematic Liquid. Phys. Rev. Lett..

[3] Alben R. (1973). Phase Transitions in a Fluid of Biaxial Particles. Phys. Rev. Lett..

[4] Golo V.L., Kats E.I., Sevenyuk A.A., Sinitsyn D.O. (2013). Twisted quasiperiodic textures of biaxial nematic liquid crystals. Phys. Rev. E.

[5] Luckhurst G.R., Sluckin T.J. (2014). Biaxial Nematic Liquid Crystals: Theory, Simulation and Experiment.

[6] Lehmann M., Görtz V. (2014). Handbook of Liquid Crystals, 11 Design of Biaxial Nematic Mesogens, Nematic and Chiral Nematic Liquid Crystals. Part III. Discotic, Biaxial and Chiral Nematic Liquid Crystals.

[7] Tschierske C., Photinos D.J. (2010). Biaxial Nematic Phases. J. Mater. Chem..

[8] Pickena S.J., Dingemansb T.J., Madsenc L.A., Francescangelid O., Samulski E.T. (2012). Uniaxial to biaxial nematic phase transition in a bent-core thermotropic liquid crystal by polarising microscopy. Liq. Cryst..

[9] Figueiredo Neto A.M., Salinas S.R.A. (2005). The Physics of Lyotropic Liquid Crystals: Phase Transitions and Structural Properties.

[10] Radley K., Saupe A. (1978). Cholesteric States of Micellar Solutions. Mol. Phys..

[11] Acimis M., Reeves L.W. (1980). A Type II Aqueous Cholesteric Lyomesophase. Can. J. Chem..

[12] Figueiredo Neto A.M., Galerne Y., Liébert L. (1985). Cholesterization of a Biaxial Nematic Lyomesophase Studied by X-Ray Diffraction and Optical Microscopy. J. Phys. Chem..

[13] Reis D., Akpinar E., Figueiredo Neto A.M. (2013). Effect of Alkyl Chain Length of Alcohols on Cholesteric Uniaxial to Cholesteric Biaxial Phase Transitions in a Potassium Laurate/Alcohol/Potassium Sulfate/Water/Brucine Lyotropic Mixture: Evidence of a First-Order Phase Transition. J. Phys. Chem. B.

[14] Brand H.R., Pleiner H. (1985). Cholesteric to Cholesteric Phase Transitions in Liquid Crystals. J. Phys. Lett..

[15] Alcantara M.R., de Melo M.V.M.C., Paoli V.R., Vanin J.R. (1983). New cholesteric and nematic lyotropic mesophases from di-sodium N-lauroyl-aspartate. Mol. Cryst. Liq. Cryst..

[16] Akpinar E., Giesselmann F., Acimis M. (2013). Effect of micelle size and intermicellar distance on the chirality transfer in the intrinsic lyotropic cholesteric phases. Liq. Cryst..

[17] De Gennes P.G., Pros J. (1993). The Physics of Liquid Crystals.

[18] Galerne Y., Marcerou J.P. (1983). Temperature behavior of the order-parameter invariants in the uniaxial and biaxial nematic phases of a lyotropic liquid crystal. Phys. Rev. Lett..

[19] Born M., Wolf E. (1980). Principles of Optics.

[20] Berejnov V., Cabuil V., Perzynski R., Raikher Y. (1998). Lyotropic System Potassium Laurate/1-Decanol/Water as a Carrier Medium for a Ferronematic Liquid Crystal: Phase Diagram Study. J. Phys. Chem. B.

[21] Du X., Hlady V. (2002). Monolayer formation on silicon and mica surfaces rearranged from *N*-hexadecanoyl-l-alanine supramolecular structures. J. Phys. Chem. B.

[22] Lu X., Zhang Z., Liang Y. (1996). Bilayer formation in dilute aqueous solution from monoalkylethylenediamine. Langmuir.

[23] Figueiredo Neto A.M., Galerne Y., Levelut A.M., Liébert L. (1985). Pseudo-lamellar ordering in uniaxial and biaxial lyotropic nematics: A synchrotron X-ray diffraction experiment. J. Phys. Lett..

[24] Deutsch M. (1991). Orientational order determination in liquid crystals by X-ray diffraction. Phys. Rev. A.

[25] Bordes R., Holmberg K. (2011). Physical chemical characteristics of dicarboxylic amino acid-based surfactants. Coll. Surf. A.

[26] Hunter R.J. (1993). Foundations of Colloid Science.

[27] Trew V.C.G. (1953). The Diamagnetic Susceptibility of some Alkyl Benzenes and Higher Aliphatic Hydrocarbons. Trans. Faraday Soc..

[28] Wendler K., Thar J., Zahn S., Kirchner B. (2010). Estimating the Hydrogen Bond Energy. J. Phys. Chem. A.

[29] Jungermann E., Gerecht J.F., Krems I.J. (1956). The preparation of long chain N-acylamino acids. J. Am. Chem. Soc..

[30] Varaşteanu D., Piscureanu A., Chican I.E., Corobea M.C. (2011). Aspects regarding the synthesis and surface properties of some glycine based surfactants. U.P.B. Sci. Bull. B.

